# 3D Bioprinting of Multi-Material Decellularized Liver Matrix Hydrogel at Physiological Temperatures

**DOI:** 10.3390/bios12070521

**Published:** 2022-07-13

**Authors:** Vamakshi Khati, Harisha Ramachandraiah, Falguni Pati, Helene A. Svahn, Giulia Gaudenzi, Aman Russom

**Affiliations:** 1 Science for Life Laboratory, Division of Nanobiotechnology, Department of Protein Science, KTH Royal Institute of Technology, 17165 Solna, Sweden; khati@kth.se (V.K.); helene.andersson.svahn@scilifelab.se (H.A.S.); giulia.gaudenzi@ki.se (G.G.); 2 Biopromic AB, 17165 Solna, Sweden; harishabio@gmail.com; 3 Department of Biomedical Engineering, Indian Institute of Technology Hyderabad, Kandi 502285, India; falguni@bme.iith.ac.in; 4 Department of Global Public Health, Karolinska Institute, 17165 Solna, Sweden; 5 AIMES—Center for the Advancement of Integrated Medical and Engineering Sciences, Karolinska Institute and KTH Royal Institute of Technology, 11428 Stockholm, Sweden

**Keywords:** decellularized liver matrix bioink, bioprinting at physiological temperatures, cytocompatible crosslinking, robust bioink, viscoelasticity

## Abstract

Bioprinting is an acclaimed technique that allows the scaling of 3D architectures in an organized pattern but suffers from a scarcity of appropriate bioinks. Decellularized extracellular matrix (dECM) from xenogeneic species has garnered support as a biomaterial to promote tissue-specific regeneration and repair. The prospect of developing dECM-based 3D artificial tissue is impeded by its inherent low mechanical properties. In recent years, 3D bioprinting of dECM-based bioinks modified with additional scaffolds has advanced the development of load-bearing constructs. However, previous attempts using dECM were limited to low-temperature bioprinting, which is not favorable for a longer print duration with cells. Here, we report the development of a multi-material decellularized liver matrix (dLM) bioink reinforced with gelatin and polyethylene glycol to improve rheology, extrudability, and mechanical stability. This shear-thinning bioink facilitated extrusion-based bioprinting at 37 °C with HepG2 cells into a 3D grid structure with a further enhancement for long-term applications by enzymatic crosslinking with mushroom tyrosinase. The heavily crosslinked structure showed a 16-fold increase in viscosity (2.73 Pa s^−1^) and a 32-fold increase in storage modulus from the non-crosslinked dLM while retaining high cell viability (85–93%) and liver-specific functions. Our results show that the cytocompatible crosslinking of dLM bioink at physiological temperatures has promising applications for extended 3D-printing procedures.

## 1. Introduction

Advancements in tissue engineering are in dire need of 3D-fabricated structures that precisely position and design the native microarchitecture of intricate tissues. Bioprinting has emerged as a powerful technique to deliver living cells embedded in a biomaterial in an organized pattern to build an intricate 3D structure layer by layer [1,2]. It offers advantages in terms of high repeatability, controllability, throughput, and positioning of multiple cells simultaneously [3,4]. However, proof-of-principle studies with 3D printing have been restricted to simple tissues such as skin and cardiac tissue [5,6], whereas heterogeneous and complex organs, such as the liver, are still challenging to engineer due to biomaterial limitations. This realization has fuelled advancements in liver-specific biomaterials for 3D printing to closely reconstitute the liver-specific extracellular matrix composition, microarchitecture, and functionality, which are critical for creating a biologically relevant liver model for applications such as drug screening/toxicity testing, in vitro studies, and organ transplantation [7].

An essential aspect of bioprinting is the choice of a printable hydrogel or “bioink” as it contains supportive cell media that directly influence the physical and biomechanical characteristics of the fabricated structure [8]. Apart from printability, these bioinks must have properties such as bioactivity, biocompatibility, shape fidelity, and the ability to maintain in vivo liver-like functions and morphology [9]. Many natural biomaterials such as alginate [10,11], collagen [12,13], and gelatin [14,15] have been previously used for extrusion-based bioprinting owing to their inherent bioactivity and excellent biocompatibility [1]. Gelatin is available in a fantastic range of viscosities and molecular weights in various functionalized forms as a rheology enhancer with either UV crosslinking or thermal crosslinking [8,16,17]. Synthetic biomaterials, on the other hand, such as poly(ethylene glycol)diacrylate [18] (PEGDA) and pluronic acid [19] have unmatched tailorability, robustness, reproducibility, and gelation kinetics with compromised biochemical features [20]. As one single biomaterial cannot fulfil all the requirements, a combination of these materials provides a more holistic approach [21,22]; however, all these materials either totally lack the native extracellular matrix (ECM) component [23] or do not entirely mimic the optimal ratios of different bioactive proteins present in a specific tissue, such as liver [24,25]. To improve these limitations, decellularized extracellular matrix as bioinks has gained support as a matrix material for its superiority in tissue-specific components, such as collagen, glycosaminoglycans (GAGs), and growth factors involved in cell signaling [2,26,27,28,29]. The ECM has a dynamic interaction between its unique microenvironment with an array of proteins for structural support and the resident cells compared to the currently used biomaterials [26,30]. Tissue specificity is vital in liver tissue engineering for promoting and preserving the proliferation, differentiation, functions, and maturation of liver cells [27,31]. This has increased the popularity of decellularized liver matrix, which is a game-changer for biomimetic bioinks [3,32,33]. However, despite its unparalleled benefits, its application as a bioink is still challenging due to poor mechanical properties and rapid biodegradation [34]. To compensate for these shortcomings, the addition of various functional biomaterials, such as Pluronic F127 [35,36] and alginates, and crosslinking methods, such as thermal, chemical, and Ultraviolet (UV), have been investigated for extrusion bioprinting [37]. However, these crosslinking methods were adopted for the added biomaterials rather than for the liver dECM, which is crucial for improving the overall viscoelastic properties and long-term applications. Moreover, all the mentioned studies on natural, synthetic, and dECM-based biomaterials did not conduct the bioprinting process at 37 °C. This can damage or modify cells during an extended printing process, where they are away from their cell culture conditions. Thus, a mechanically strong dECM-based bioink printable under physiological conditions may benefit encompassing cells and their survival.

**Figure 1:**
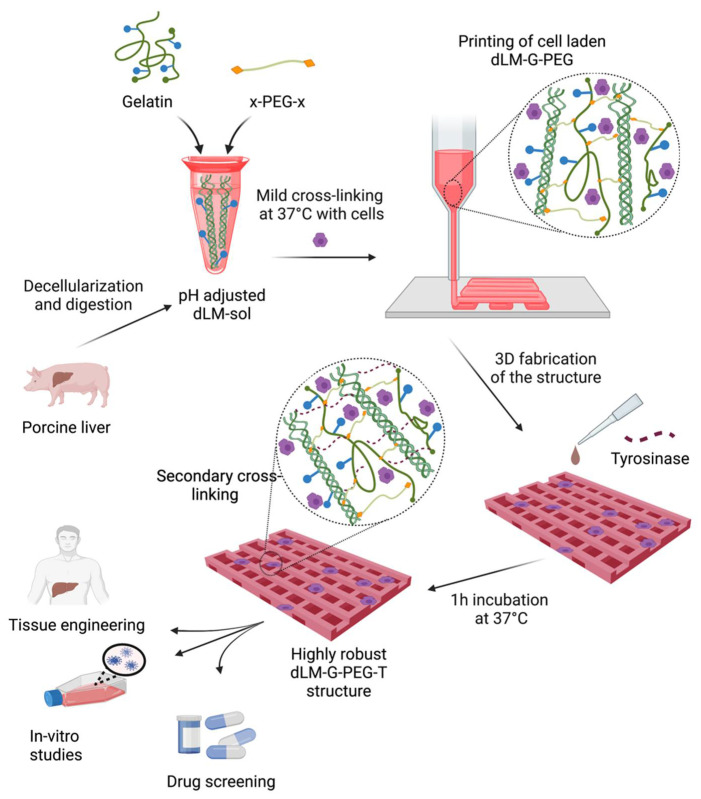
Schematic of the development of the dLM-G-PEG construct and post-printing crosslinking with tyrosinase. Liver tissue is decellularized and digested to form pH-adjusted dLM-sol. Gelatin and an x-PEG-x crosslinker are added followed by the addition of cells. This formulation is crosslinked at 37 °C and then printed into a grid structure. Mushroom tyrosinase is added to the 3D printed structure and incubated for 1 h at 37 °C in the cell incubator. The final structure is heavily crosslinked and has application in tissue engineering, in vitro studies, and drug screening.

Herein, we develop a novel dLM (decellularized liver extracellular matrix)-based bioink with gelatin as a rheology enhancer that is jointly crosslinked chemically by succinimidyl valerate-polyethylene glycol- succinimidyl valerate (x-PEG-x, x = succinimidyl valerate) (Figure 1). PEG is an FDA-approved biomaterial that utilizes the typical functional group [38] (amines) in both dLM and gelatin to form a robust bioink (dLM-G-PEG, G = gelatin) via the cytocompatible gelation method. The 3D bioprinting is conducted under physiological conditions at 37 °C with HepG2 cells to develop a four-layer grid structure. Mushroom tyrosinase was used to crosslink and further improve the mechanical properties of the 3D construct by enzymatic crosslinking of the available tyrosine residues of dLM and gelatin [8,39] (dLM-G-PEG-T, T = mushroom tyrosinase). We investigated the changes in viscoelastic properties and crosslinking using rheology and by quantifying the concentration of free amines, respectively. We used HepG2 cells due to their similarity in functions to the native liver [40] and observed their cellular response, specifically the proliferation, albumin secretion, and gene expression within the dLM-G-PEG-T construct at distinct time points for seven days.

## 2. Materials and Methods

Porcine liver was bought from a local slaughterhouse. Gelatin powder (Porcine skin, Type A) was purchased from Sigma-Aldrich AB, St. Louis, MO, USA, succinimidyl valerate-PEG-succinimidyl valerate (MW 5000) from Laysan bio-Inc, Arab, AL, USA and mushroom tyrosinase (25KU, ≥1000 unit/mg solid) from Sigma-Aldrich AB. Unless stated, all other reagents were procured from Sigma-Aldrich Sweden AB. Details about the other reagents are given with the following methods.

### 2.1. Decellularization of Liver

The protocol for liver decellularization was modified from a previously reported work [2,41]. Frozen liver tissue was thawed to 4 °C and cut into small pieces of 1 mm thickness. Next, the chopped tissue was washed with DI water to remove excess cell debris, followed by sodium dodecyl sulfate (powder, ≥99%) treatment with increasing concentrations from 0.1% to 1% (made in DI water). After 2–3 days, the tissue was rewashed with 1% phosphate-buffered saline (PBS) solution for 24 h and treated with Triton X-100 (liquid) for 30 min. Then, the tissue was rewashed with PBS and sterilized with 0.1% peracetic acid and 4% ethanol for 4 h. The decellularized tissue was washed several times with PBS and DI water for the next 24 h. Lastly, it was lyophilized and stored for the long term at −20 °C.

### 2.2. Biochemical Analysis of dLM

To check the efficiency of decellularization, ECM components such as collagen and GAGs were each characterized in 10 mg of native and decellularized tissue. A hydroxyproline assay kit (ab222941, Abcam, Cambridge, UK) was used to quantify collagen according to the manufacturer’s protocol. Briefly, the dried tissues were solubilized in 100 μL of sodium hydroxide solution (NaOH) at 120 °C for 1 h, followed by the absorbance measurement of the hydroxyproline standard and the samples at 560 nm. The sample readings were applied to the standard curve to obtain the amount of hydroxyproline. The concentration of collagen was normalized to the dry weight of the tissue. The GAGs in the tissues were estimated using a 1,9-dimethylmethylene blue (DMMB assay) solution to quantify the sulfated glycosaminoglycans using chondroitin sulfate A as a reference at a wavelength of 540 nm [42]. 

DNA analysis was performed using a commercially available DNA extraction kit (PureLinkTM, Genomic DNA mini kit). Briefly, the amount of total DNA in 2 mg of dry liver samples before and after decellularization was measured in a Nanodrop (N60, Implen, München, Germany) at 260 nm. 

For histology, the native and decellularized liver tissues were fixed in formalin solution (4%), washed with distilled water, and embedded in the OCT compound. Next, the tissue was sectioned in the cryotome, stained with Hematoxylin and Eosin, and checked under a microscope [2].

### 2.3. Preparation of dLM Formulations

The lyophilized liver tissue was crushed with a mortar and pestle into a fine powder. An appropriate amount of the tissue was weighed and digested with pepsin in 0.5 M acetic acid [2]. The quantity of pepsin added was 10% of the dry weight of the tissue. The tissue was solubilized within 48–72 h into a 3% dLM solution (designated as dLM-sol), with a pH value of around 3. The pH of dLM-sol was adjusted to a 7–7.4 value with cold 10 M NaOH solution and designated as pH-adjusted dLM-sol. To prepare the bioink, 10–12% warm gelatin solution (in water) was mixed with the pH-adjusted dLM-sol at a volume ratio of 1:5 (designated as dLM-G). Immediately, x-PEG-x was introduced as a crosslinker at a concentration of 14.4 mg/mL of the dLM-G mix and crosslinked at 37 °C for 1 h. The dLM-G-PEG bioink was ready to be used for cell encapsulation and bioprinting into a 3D grid construct. Instantly, tyrosinase was added dropwise in a concentration of 500 units per ml to the 3D structure and further crosslinked for about 1 h in a cell incubator at 37 °C and designated as dLM-G-PEG-T. Detailed descriptions of all the formulations are provided in Supplementary Table S1.

### 2.4. Characterization of the dLM Formulations

The liver bioink was characterized physically by rheology, and the crosslinking in the bioink was determined by Tri-nitro benzene sulfonic acid (TNBS) assay. To evaluate the crosslinking and printability of the dLM bioink, a comparative analysis was performed between different formulations at 37 °C, as shown in Table S1 (Supplementary Materials). The rheological properties were investigated in TA Instruments Discovery Hybrid rheometer (New Castle, PA, USA) with a 25 mm parallel plate. The viscosity of the samples was analyzed with a steady shear sweep at 37 °C. Gelation kinetics of dLM-G-PEG and dLM-G-PEG -T were studied with a temperature sweep for 3000 s with continuous complex modulus measurements at 37 °C. In the amplitude sweep, dLM-G-PEG and dLM-G-PEG -T were evaluated for oscillation strain ranging from 0.1–100% at a constant frequency of 1 Hz. A dynamic frequency sweep was performed from 0.1 to 100 rad s^−1^ at 1% strain to assess the frequency-dependent storage and loss modulus for dLM-G-PEG and dLM-G-PEG-T. 

TNBS assay was slightly modified from a previously used protocol [43]. To summarize, 1.8 mg of dry tissue samples were treated with 0.05% TNBS (5% *w*/*v*, Picrylsulfonic acid solution) and 4% NaHCO_3_ (1 M, pH 8.5, Fisher Scientific) solution for 2 h at 40 °C. Further, these samples were hydrolyzed at 60 °C for 90 min and the absorbance was measured at 320 nm. 

Lastly, scanning electron microscopy was performed to determine the topography of the pH-adjusted dLM and dLM-G-PEG bioink using the Hitachi TM-1000 scanning electron microscope (Hitachi, Tokyo, Japan). 

### 2.5. Cell Culture, Maintenance, and Encapsulation in dLM-G-PEG Bioink

The human hepatocellular carcinoma cell line HepG2 (Sigma Aldrich Sweden AB) was used to check the biocompatibility of the dLM-G-PEG bioink during the gelation and printing process. HepG2 cells were cultured according to the manufacturer’s protocol, with Eagle’s minimal essential medium (MEM, 11095080), 10% fetal bovine serum (FBS, A3160502), and penicillin-streptomycin (10,000 U mL^−1^, 15140122). Cells were maintained in culture and passaged every 2–3 days at about 80–90% confluency. Once at proper confluency, cells were used for encapsulation. Briefly, HepG2 cells were dissociated from the culture plate with trypsin-EDTA (0.25% solution, Sigma-Aldrich AB) and centrifuged at 300× *g* for 4–5 min. The collected HepG2 cells were resuspended in 10% FBS. To sustain the same osmotic pressure in dLM-G-PEG, 10× concentrated MEM (21430020, Gibco) was added (1/10th volume) and the collected HepG2 cells in 10% FBS, were mixed thoroughly with the acellular bioink. HepG2 cells were used in the dLM-G-PEG at a concentration of 4 to 5 × 10^6^ cells per ml. The prepared bioink with HepG2 cells was crosslinked for 1 h at 37 °C and loaded into a sterilized syringe for bioprinting while maintaining the temperature. Cell culture reagents for HepG2 cell culture and maintenance were obtained from Thermofisher Scientific.

### 2.6. Bioprinting of dLM-G-PEG Construct

A CellInk bioprinter with a temperature-controlled printing nozzle was used for printing a 6-layer grid structure with controllable pneumatic pressure. The dLM bioink was dispersed with a sterile nozzle with a diameter of 0.4 mm at 30–40 kPa pneumatic pressure and 4–6 mm s^−1^ printing speed. The 3D grid structure was designed with dimensions of 10 × 10 × 2.4 mm in Autodesk Fusion 360 and uploaded as a G-code in the bioprinter. Some parameters were optimized continuously during printing such as pneumatic pressure. The dLM-G-PEG printed structure was further crosslinked with tyrosinase for 1 h and analyzed for various changes in parameters such as line width, space between printed lines, dimensions of the structure, and volumetric changes. This acellular dLM-G-PEG-T construct was set aside and observed for 3D printed dimensions and long-term stability. Microscopy analyses were performed on day 21. 

Another 4-layer grid structure was printed similarly with HepG2 cells, crosslinked with tyrosinase for 1 h, and washed with PBS for 5–10 min. Next, the whole structure was submerged in 2 mL of HepG2 cell complete medium as previously described and placed at 37 °C with 5 % CO_2_. The media was replaced after every 24 h. This dLM-G-PEG-T structure was observed for 7 days for biocompatibility, viability, and liver-specific functions.

### 2.7. Live Dead Assay and cell Proliferation

Cell viability and proliferation were investigated on days 1, 3, and 7. Fluorescence staining was conducted to assess the live cells using Calcein AM (2 μM mL^−1^) and dead cells using Ethidium homodimer-1 (4 μM mL^−1^). In summary, the scaffolds were washed with PBS and stained for 30 min in cell culture conditions. A confocal microscope was used to capture all the images with a 10× objective and a 2.5× objective at different time points. A control group of collagen type 1 rat tail (C3867, Sigma-Aldrich AB) with HepG2 embedded cells was used to compare the cell viability and proliferation.

The proliferation of the HepG2 cells at the same time points was assessed using Alamar blue assay with slight modifications. The cell-loaded dLM-G-PEG-T scaffold was washed twice with PBS and Alamar was added with the cell culture media at a ratio of 1:10. The scaffold was again placed at 37 °C for 3 h and the supernatant was collected in a 96-well plate. The redox indicator in Alamar changes the color of the blue Alamar (oxidized form) to pink (reduced form), showing cell proliferation. The samples were read at fluorescence intensity of 540 nm excitation and 590 nm emission against a blank control [44,45].

### 2.8. Liver Functionality and Gene Expression Analysis with RT-PCR

Culture medium from HepG2 cells on days 1, 3, and 7 was collected to test the levels of albumin from 3D printed dLM-G-PEG-T and the collagen control. The Human Albumin ELISA quantification kit (ab179887, Abcam) was used according to the manufacturer’s protocol. 

To evaluate the mRNA gene expression, total RNA was collected from HepG2 cells embedded in the dLM-G-PEG-T scaffold at the same time points as above using RNeasy Mini Kit (Qiagen), followed by cDNA synthesis using High-Capacity cDNA Reverse Transcription Kit (4368814, ThermoFisher Scientific, Waltham, MA, USA). RT-qPCR was conducted using SYBR™ Green PCR Master Mix (4309155, ThermoFisher Scientific) and the StepOnePlus PCR instrument (Applied Biosystems, Waltham, MA, USA). The raw data were processed using the StepOnePlus instrument’s software. The results obtained for the mRNA expression level of four liver-specific genes, AFP (Alpha-Fetoprotein), ALB (Albumin), KRT19 (Keratin 19), and MKI67 (Marker of proliferation Ki-67), were subsequentially normalized to the mRNA expression level of the housekeeping glyceraldehyde-3-phosphate dehydrogenase (GAPDH) and analyzed using GraphPad Prism (version 9).

### 2.9. Statistical Analysis

All measurements are carried out in triplicate and expressed as the standard error of the mean. The data and statistical analysis were performed in GraphPad Prism version 9. One-way ANOVA with Bonferroni correction was used to present statistical significance. The difference was statistically significant with *p* < 0.05, 0.01, 0.001 and 0.0001 represented by *, **, *** and **** respectively.

## 3. Results and Discussions

### 3.1. Preparation and Crosslinking of dLM

**Figure 2:**
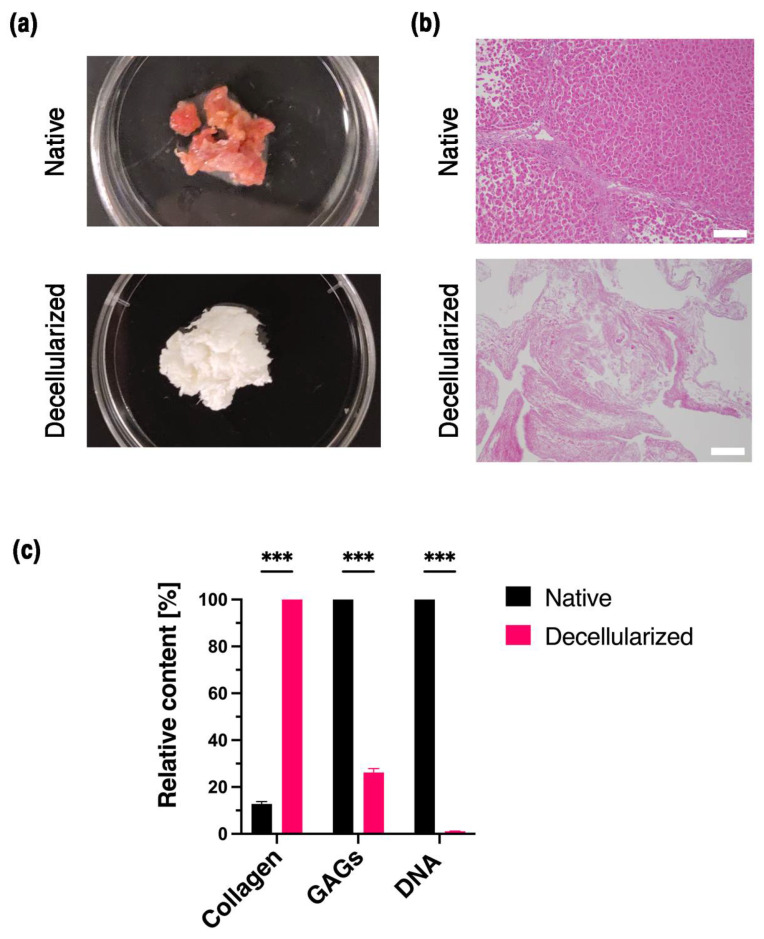
Decellularization and the biochemical analysis of liver tissue. (**a**) Optical image of native and decellularized liver tissue and (**b**) microscopic image of native and decellularized liver tissue (scale bar 100 µm). (**c**) Liver ECM components collagen and GAGs with the DNA content of native and decellularized tissue. All experiments were conducted in triplicate. Error bars display the standard error of the mean (*** *p* < 0.001).

The decellularization process aimed to retain the maximum ECM components, specifically collagen. Liver decellularization is an extensive process as the liver contains most of the cell population compared to the ECM [46]. The liver was efficiently decellularized (Figure 2a) using sodium dodecyl sulfate (SDS) and Triton-X 100 using a few previously published methods [2,41]. Successful decellularization was evaluated using a DNA quantification assay at a 98.6% reduction of DNA with 32.1 ± 4.85 ng per mg remaining in the decellularized liver. This value is below the accepted threshold of 50 ng per mg of DNA level in dry tissue. Still, to further confirm the results, hematoxylin and eosin (H&E) staining was performed to reveal the removal of cell and cell debris after decellularization (Figure 2b). 

Primary liver ECM components such as collagen and sulfated glycosaminoglycans (GAGs) were also compared before and after decellularization (Figure 2c) with DMMB assay and Hydroxyproline assay, respectively. There was a noteworthy loss of GAGs with a ~74% reduction in the native liver and only 2.03 ± 0.17 µg per mg remaining in the decellularized tissue. GAGs are mainly localized in the cell membrane and lie within the ECM as they are associated with growth factors that stimulate cell proliferation and differentiation [47,48]. The decellularization protocol caused a significant loss of GAGs along with a disruption to the natural orientation of the ECM fibers. On the contrary, the collagen content increased statistically after decellularization to 29.05 ± 0.52 µg per mg in the decellularized tissue. This is due to the low percentage of cellular components remaining in the decellularized liver compared to the ECM [41]. This can be explained as the dry weight of the native liver is mainly due to the cell population, which is included in the hydroxyproline assay resulting in a low collagen per total dry weight. Thus, the hydroxyproline assay fails to provide the actual value of the collagen in native tissue, making the comparison between native and decellularized tissue ambiguous.

**Figure 3:**
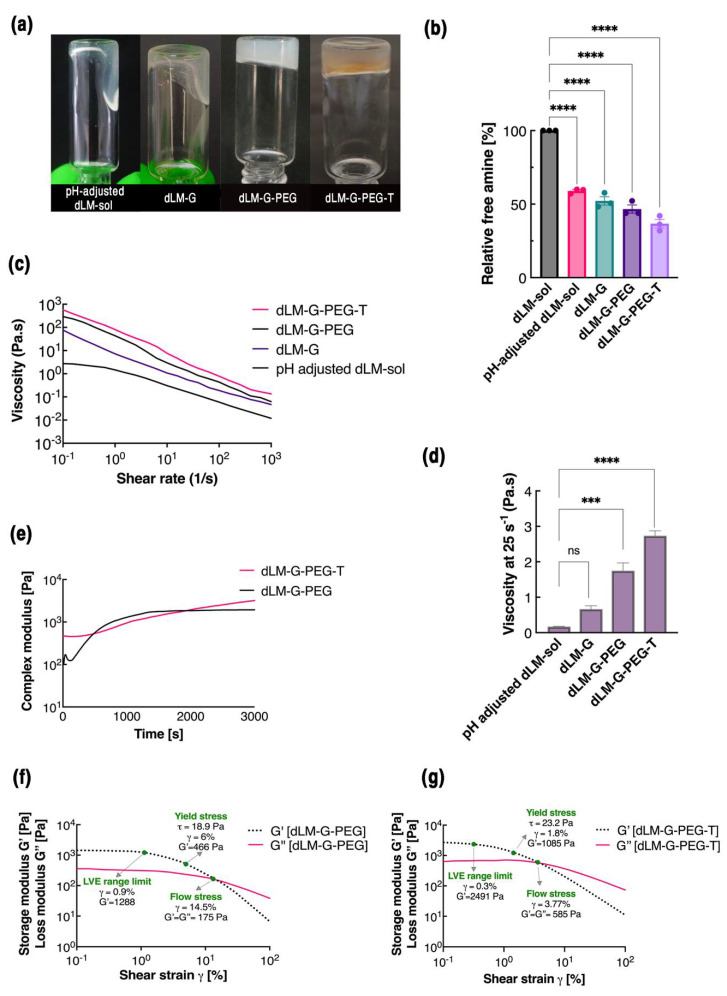
Characterization of dLM formulations (**a**) visually for pH-adjusted dLM-sol, dLM-G, dLM-G-PEG bioink, and dLM-G-PEG-T at 37 °C and (**b**) TNBS assay with decreasing free amines relative to dLM-sol (**** *p* < 0.0001) Rheological properties at 37 °C with (**c**) viscosity at increasing shear rates and (**d**) comparison of viscosities of different formulations at 25 s^−1^ (*** *p* < 0.001 and **** *p* < 0.0001). (**e**) Gelation kinetics of dLM-G-PEG bioink and dLM-G-PEG-T. Oscillatory amplitude sweep analyzed at 1 Hz angular frequency to estimate the mean yield stress (n = 3), linear viscoelastic (LVE) range, and flow stress to analyze storage (G’) and loss (G’’) modulus from 0.1 to 100% strain for (**f**) dLM-G-PEG and (**g**) dLM-G-PEG-T.

Here, our objective is the development of a dLM bioink printable at physiological temperatures with enhanced rheological properties and stable crosslinking. The first step for formulating a hydrogel was the enzymatic digestion of decellularized liver with acetic acid to form a free-flowing dLM-sol at a concentration of 3% (Supplementary Materials, Figure S2) with a pH of around 3. dLM-sol is the crude form of the pepsin-digested liver tissue, which is not sensitive to any temperature variations. This solution was then adjusted to a physiological pH value of 7 (pH-adjusted dLM-sol) at a temperature below 10 °C (Figure 3a), followed by the addition of 10% gelatin to improve the rheological behavior (dLM-G). With gelatin concentrations lower than 10–12%, a higher volume of the gelatin solution was required to be added with pH-adjusted dLM-sol to provide an excellent viscoelastic property. This would increase the water content of the end formulation, resulting in free-flowing dLM-G as observed with tube inversion and thus was not suitable for further evaluations (Supplementary Materials, Figure S3). Finally, a 10–12% gelatin concentration was tested at lower volumes with pH-adjusted dLM-sol to improve the mechanical properties of the dLM-G (Figure 3a) formulation. The crosslinker x-PEG-x was promptly added to crosslink the available amine groups. The concentration of x-PEG-x was kept low to create a robust bioink, easily printable at a low pneumatic pressure of 4–6 mm s^−1^ in the 3D printer. The optimization of the gelatin volumes with fixed x-PEG-x concentrations was evaluated (Supplementary Materials, Figure S4) to observe a specific pattern with the bioink’s viscoelasticity. Gelatin formulations with 10–12% concentration were formulated with pH-adjusted dLM with different volume ratios as shown in Supplementary Materials, Figure S4a. All the results were based on their behavior with tube inversion. At low volume ratios of around 1:10, more brittle and easily breakable formulations were formed, which also failed to 3D print (Supplementary Materials, Figure S4b); however, with a higher gelatin volume ratio of 1:2, softer formulations were formed that did not pass the tube inversion test. The formulations were designated as either ‘robust’ if they maintained shape, ‘soft’ if they spread easily, or ‘brittle’ if they broke easily (Supplementary Materials, Table S2). The robust formulations formed were between a 1:4 and 1:6 volume ratio of gelatin that maintained its shape when spread with a spatula and injected on a surface (Supplementary Materials, Figure S5). Based on our observations, a 1:5 volume ratio seemed the most robust to be selected for further analysis (Figure 3a). The resulting bioink was designated as dLM-G-PEG and formed a soft gel in 1 h at 37 °C before cell encapsulation (Figure 3a). For extrusion bioprinting, instant gelation helps shape fidelity and increases the viscosity of the printed filaments. Thus, 500 U mL^−1^ of tyrosinase was used, based on a previous study [8], as a secondary crosslinker to enhance the robustness. The brownish stain in the bioink is due to tyrosinase (Figure 3a).

### 3.2. Characterization of dLM Formulations

With all the essential components of dLM bioink prepared, a comparative study was performed for different acellular formulations to determine printability. Each formulation was validated to decide on its crosslinking and viscoelastic properties. The effect of the cross-linking strategy was calculated as residual free amine groups in the TNBS assay. It revealed the number of available amine groups that did not participate in crosslinking (Figure 3b) relative to dLM-sol, which was assumed to contain 100% of the free amines with no crosslinking. A higher concentration of the free amines in pH-adjusted dLM-sol followed by dLM-G demonstrated a low degree of crosslinking. However, dLM-G-PEG and dLM-G-PEG-T demonstrated 46.6% (±2.78) and 36.7% (±2.92) of the free amine groups, respectively. This justifies the application of loosely crosslinked dLM-G-PEG as a bioink as well as further improvements in crosslinking in dLM-G-PEG-T. 

To further verify the suitability of the formulations, rheological properties were measured at 37 °C after gelation to determine the viscoelasticity, flow behavior, and gelation kinetics to mimic the 3D printing process. All the formulations demonstrated shear-thinning behavior with a drop in the viscosity with the increasing shear rate in the measured range (Figure 3c). This is called non-Newtonian behavior, crucial in conserving encapsulated cells to generate lower shear stress during the extrusion printing through a small diameter nozzle. The shear rate generated through a 410 μm nozzle during the printing process was calculated to be between 16.35 s^−1^ and 34.5 s^−1^, which can be correlated to the viscosities. This value was correlated to the viscosity of the dLM-G-PEG in the range of 2.08 Pa s^−1^ to 1.5 Pa s^−1^. At a 25 s^−1^ shear rate, the viscosity of pH-adjusted dLM-sol is 11× lower than the dLM-G-PEG bioink implying a fragile nature without crosslinking. With such low viscosity, the application of pH-adjusted dLM-sol would be extremely limited for 3D tissue structures, which proves the necessity for additional reinforcement. After adding gelatin, the viscosity of dLM-G drastically improves but it is still 2.6× lower than the dLM-G-PEG, implying the importance of x-PEG-x to crosslink both components, i.e., dLM and gelatin. The viscosity of dLM-G-PEG was around 1.75 Pa s^−1^, which is an intermediate value among all the formulations. Secondary crosslinking by tyrosinase further increases the viscosity to 2.73 Pa s^−1^ with a 1.6-fold increase from dLM-G-PEG and a 16-fold increase from pH-adjusted dLM-sol, suggesting a highly crosslinked biomaterial. The information on the viscosity behavior complements the TNBS results, with pH-adjusted dLM-sol having the lowest viscosity and dLM-G-PEG-T having the highest viscosity. A lower viscosity may result in weak extruded filaments that collapse easily and do not retain shape after printing. 

The storage (G’) and loss (G’’) modulus of all the formulations under oscillatory conditions exhibited a typical elastic effect with a higher storage modulus than loss modulus (Supplementary Materials, Figure S6), which is crucial for high shape fidelity after extrusion. Both G’ and G’’ for dLM-G-PEG and dLM-G-PEG-T were stable throughout the tested frequency range with the highest modulus illustrated by dLM-G-PEG-T followed by dLM-G-PEG, and as a continuation of the previous results, pH-adjusted dLM-sol and dLM-G also demonstrated the lowest values for modulus. The dLM-G-PEG-T showed a G’ of 1928 Pa, which is 32× higher than the pH-adjusted dLM-sol (Supplementary Materials, Table S3). Based on these observations, hereafter, only dLM-G-PEG was validated as a desirable formulation for the printing process at 37 °C and dLM-G-PEG-T as a robust post-printing formulation for the remainder of the experiments. 

Other important rheological parameters to impact the printing process are the gelation kinetics and yield stress, τ_y_. The gelation kinetics of dLM-G-PEG and dLM-G-PEG-T were evaluated at 37 °C (Figure 3d). As soon as the dLM-G-PEG reached 37 °C, a sudden increase in the complex modulus was observed, indicating immediate gelation, and crosslinked bioink was formed within 30 min. A plateau after 30 min of gelation indicated a fully crosslinked bioink. On the contrary, dLM-G-PEG-T demonstrated contrasting behavior with a considerable increase in the modulus until the end of the experiment, resulting in a stiffer gel. Thus, the presence of tyrosinase increased the mechanical properties of the comparatively softer dLM-G-PEG bioink for long-term stability.

Additionally, the oscillatory amplitude sweep was performed between 0.1% and 100% strain with the sole purpose of obtaining the equilibrium shear modulus and linear viscoelastic (LVE) region. The τ_y_ was determined by calculating the oscillatory stress from the applied strain using Trios software. The oscillatory amplitude sweep of dLM-G-PEG demonstrated a plateau or LVE region of G’ between a 0.1 and 0.97% strain (Figure 3d). This is the reason for choosing a 1% strain as the standard to conduct the oscillatory frequency experiment without irreversible deformation. We evaluated that the τ_y,_ which is the minimum stress necessary to initiate the bioink flow through the nozzle, was around 18.9 Pa. Below this value, any deformation in the structure is small and reversible. It demonstrates that dLM-G-PEG exhibits a dominant elastic behavior prior to τ_y_ but with an increasing shear rate, G’ starts to drop. After reaching a strain of 14.5%, G’ and G’’ become equal with a value of 175 Pa and crossover with the dominant viscous flow. Next, the LVE value for dLM-G-PEG-T was found to be comparatively lower at between 0.1% and 0.3% (Figure 3e). The highly crosslinked dLM-G-PEG-T showed a higher τ_y_ of 23.2 Pa at a high G’ compared to the dLM-G-PEG implying better shape retention. The presence of tyrosinase may be responsible for higher yield stress in dLM-G-PEG-T. Both formulations showed elastic behavior until the yield stress but with increasing strain, a crossover point is reached where the viscous flow behavior became dominant. Higher values of τ_y_ in both dLM bioinks suggested better stackability of the filaments high up in the z-direction.

Overall, the results from the rheology study and TNBS assay demonstrated that formulations without an x-PEG-x crosslinker and tyrosinase have a lower modulus, are less viscous, and have a low degree of crosslinking. This was further supported by the SEM images of pH-adjusted dLM-sol and dLM-G-PEG bioinks that show a decrease in porosity after crosslinking, resulting in a tighter structure in the bioink (Supplementary Materials, Figure S7). It shows the improvement in the extrudability and shape fidelity of dLM with the addition of gelatin and x-PEG-x. Previous studies have shown that bioinks with relatively lower G’ tend to be more cell-friendly [49] and bioinks with a higher viscosity might exhibit more favorable printing. Also, the concentration of proteins in decellularized tissue is an essential factor that impacts rheological properties [50]. 

The gelation behavior of the dLM with gelatin in the presence of x-PEG-x is an interplay of all the ECM components taking part in the crosslinking process. Moreover, the method of decellularization is an important factor to modulate the rheological parameters as the distribution of various components differs from the adapted decellularization protocol [51]. 

We used the same decellularization protocol for the liver every time; however, with every new batch of tissue from another porcine source, dissimilar periods for completing decellularization and digestion of the liver tissue were observed. As a result, the concentration of the gelatin and x-PEG-x added to the dLM differed slightly with every new batch of liver tissue. Thus, the reproducibility of the formulation became a challenge leading to difficulties replicating the results. To avoid this, a big batch of the porcine liver was used for all the experiments to minimize the variabilities. Since the innate environment of the tissue decides the cellular functions, the dLM bioink should presumably support liver-specific cells.

### 3.3. Printing of dLM-G-PEG Bioink with HepG2 Cells

**Figure 4:**
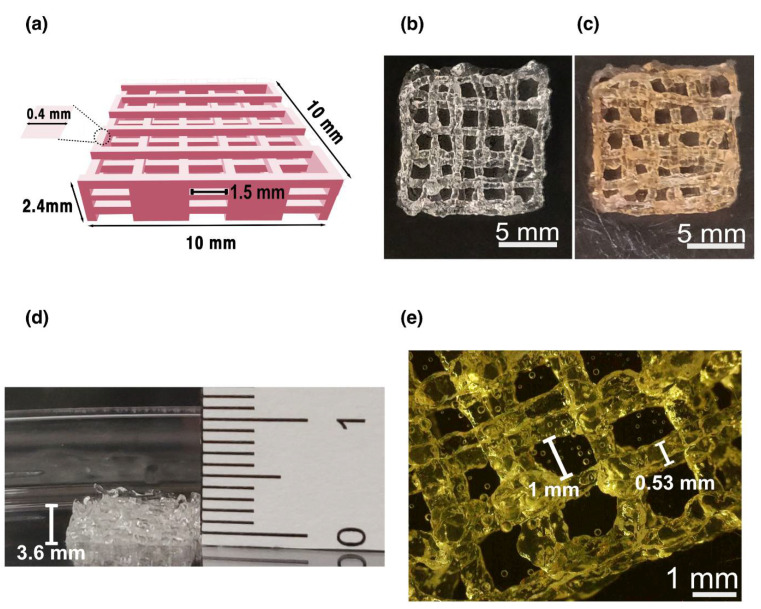
3D printed bioink constructs. (**a**) A CAD representation of the structure before bioprinting with the dimensions showing line width and line spacing. Representative images of the top view of (**b**) dLM-G-PEG and (**c**) dLM-G-PEG-T 3D bioprinted structures (scale bar, 5 mm) followed by (**d**) the side view of the construct showing height. (**e**) The microscopic image of the dLM-G-PEG bottom layer represents the line width and line spacing (scale bar, 1 mm).

We successfully fabricated a 3D porous construct from dLM-G-PEG allowing the encapsulated HepG2 cells to migrate freely to form functional tissue. A CAD grid model with 10 × 10 × 2.4 mm dimensions was printed in a CellInk bioprinter with a line width of 0.4 mm and line spacing of 1.5 mm (Figure 4a). During the extrusion process, the temperature was continuously maintained at 37 °C to obtain printability with the dLM-G-PEG and to form a cell-friendly environment within the soft-state of the dLM-G-PEG bioink in the syringe, and to obtain an easily printable filament from the nozzle (Supplementary Materials, Figure S8a,b). A self-standing grid-shaped structure with dimensions of around 11 × 11 mm (Figure 4b) was printed layer by layer and further crosslinked with tyrosinase giving its signature brown tint (Figure 4c). The height of the printed construct increased to 3.3 mm compared to the CAD model (Figure 4d). With a 0.4 mm nozzle, the line width obtained was between 0.45 and 0.55 mm in the construct, with a line spacing of between 0.9 and1.3 mm (Figure 4e) as observed in the base layer. This shows the high shape fidelity of the bottom layer after printing without any spreading due to the weight of the whole 3D structure. Thus, an overall increase in the volume of the 3D printed structure was observed with a 35–37% dimensionality increment. This might be due to the introduction of HepG2 cells with media before printing, making the hydrogel softer. During printing, the temperature was maintained at 37 °C to form a cell-friendly environment within the soft-state of the dLM-G-PEG in the syringe. Handling soft bioinks is challenging and without protocols could result in breakage, deformation, and reproducibility issues. To control these problems, immediately after printing, the complete structure was immersed in tyrosinase solution to further crosslink and prevent further deformation of the layers and was kept in the cell incubator for secondary crosslinking (Figure 4d). During this incubation, swelling in the filament width resulted in enlargement, with an increased filament width of up to 0.6 mm (Supplementary Materials, Figure S8c) [39].

The long-term stability of the dLM-G-PEG-T acellular construct in PBS was simultaneously studied for applications in tissue engineering. The construct was found to be stable for 7 days without microscopically significant changes other than more swelling of the printed filament. However, on day 21 (Supplementary Materials, Figure S8d), the constructs were slightly deformed and unstable. The constructs were visibly fragile to movements in the well plate and a part of the filament was lost while pipetting the media. Microscopic images further revealed deformed bottom filaments (Supplementary Materials, Figure S8e). Previously performed work with gelatin and dECM from other tissues has shown similar behavior of fast degradation of the bioinks, leaving behind a weak structure with voids [2,8]. Hence, a short 7-day study with HepG2 cells was conducted for further analysis in cell culture media.

### 3.4. HepG2 Proliferation and Liver-Specific Expression

**Figure 5:**
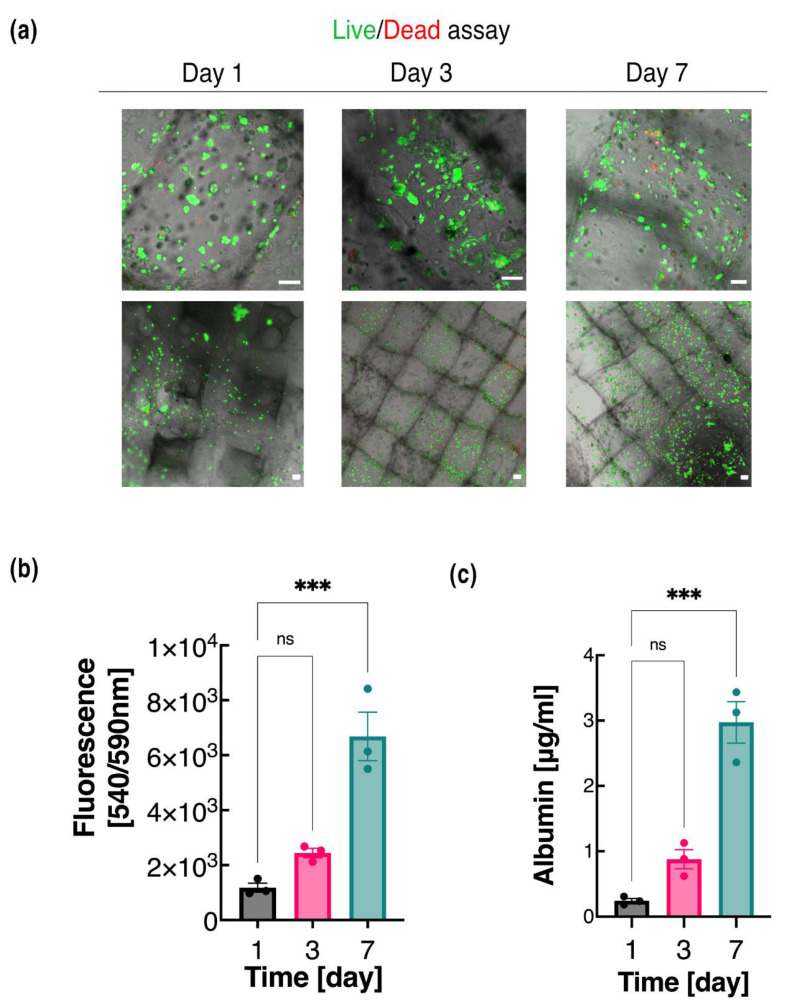
HepG2 cell proliferation embedded in dLM-G-PEG-T construct. (**a**) Live/dead images on day 1, day 3, and day 7 using 10× objective (top) and 2.5× objective (bottom) (scale bar 100 µm), (**b**) Alamar blue assay measurement of cell metabolic activity with fluorescence intensity at 540 nm excitation and 590 nm emission, and (**c**) albumin secretion measurements at various time points. All experiments were conducted in triplicate. Error bars show the standard error of the mean (*** *p* < 0.001).

The HepG2-embedded dLM-G-PEG-T construct was evaluated for cell proliferation, liver-specific functions, and gene expression analysis. Increasing cell proliferation was observed through live/dead assays with calcein AM and ethidium homodimer for 7 days with homogeneous cell distribution across the construct in 10× objective and 2.5× objective (Figure 5a). Almost no dead cells were observed on days 1 and 3, with high cell viability of 93.5% and 89.4%, respectively (Supplementary Materials, Figure S9). The cells are unevenly scattered on day 1, as visible in the 2.5× magnification image, but become evenly distributed by day 3 (Figure 5a). On day 7, cell death increased and the viability dropped to 85.8%; however, colonies of HepG2 cells were formed. Interestingly, even after 7 days of culture, there was still scope for HepG2 cells to proliferate further in the bioink. An Alamar blue assay was used simultaneously to quantify the metabolic activity of the HepG2 cells by converting the resazurin into viable cells (Figure 5b). A steady increase in fluorescence was observed for 7 days showing an increase in viable cells and no cytotoxic effect of the dLM-G-PEG-T on the cells. This indicates that dLM-G-PEG-T is biocompatible, and the shear conditions generated during extrusion with dLM-G-PEG were also cytocompatible. All these results were compared to the collagen control samples, which also showed increasing proliferation throughout (Supplementary Materials, Figure S10a,b). Thus, dLM-G-PEG-T and the collagen control provide a supportive microenvironment for the HepG2 cells. 

A more extensive characterization of liver-specific metabolic activity was observed by analyzing the albumin production, which was quite noticeable from day 1 to day 7 (Figure 5c) in the 3D printed construct. A 4-fold increase of albumin from day 1 to day 3 was observed, which jumped statistically on day 7 with a 12-fold increase from day 1. Overall, with increasing culture time, increments in albumin production visibly matched the observed cell proliferation from day 1 to day 7. However, in the collagen control sample, the albumin production shows a sluggish increase between day 3 and day 7 (Supplementary Materials, Figure S10c). Thus, 3D bioprinted dLM-G-PEG-T has a comparatively higher and more consistent production in the 3D printed dLM-G-PEG-T construct compared to the collagen control. 

**Figure 6:**
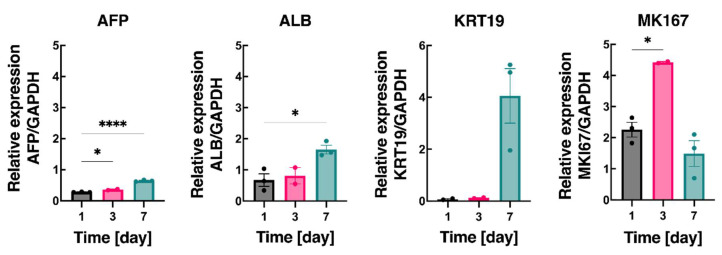
Gene expression analysis is relative to GAPDH of AFP, ALB, KRT19, and MKI67 at various time points. All experiments were conducted in triplicate. Error bars show the standard error of the mean (* *p* < 0.05 and **** *p* < 0.0001).

Lastly, we evaluated the changes in the 3D printed structure over time in terms of gene expression. We tested the mRNA levels of the characteristic hepatic markers, AFP, ALB, KRT19, and MK167 (Figure 6). The results were normalized to the reference housekeeping gene GAPDH. The mRNA levels of AFP and ALB increased moderately for 7 days. However, the mRNA levels of KRT19 were significantly lower on day 1 and day 3 but statistically increased on day 7. Furthermore, the MKI67 mRNA levels were variable and did not follow a specific trend. Taken together, these observed mRNA levels show an increasing liver-specific activity (AFP and ALB) over 7 days. However, a more comprehensive panel of liver-specific genes would provide further details about the changes in the transcriptional levels.

The overall results show the improvement in hepatic functions in the dLM-G-PEG-T construct embedded with HepG2 cells over a period of 7 days. We have formulated a cytocompatible bioink with dLM-G-PEG printable at 37 °C and provided a protocol for secondary crosslinking to enhance the mechanical properties. Tyrosinase significantly alters the properties of dLM-G-PEG for long-term analysis and cell growth. Here, we obtained a synergistic interplay through the combination of the liver-specific properties of dLM and tailorable viscoelastic properties of gelatin to fabricate a soft bioink. Moreover, x-PEG-x targets both dLM and gelatin for mild crosslinking at 37 °C allowing the addition of cells directly into the bioink immediately before crosslinking. Further improvements are required in 3D printing systems to create a uniform heating environment for temperature-sensitive bioinks for long printing processes. This way, complex architectures mimicking the liver lobules with higher resolutions can also be produced. There is a possibility to fabricate other dLM bioinks with different biomaterials using x-PEG-x as a joint crosslinking agent to create a heterogeneous structure. The stability of the printed constructs can be further improved using higher concentrations of tyrosinase. This study can find further applications in studying cancer models representative of tumors, spheroid systems, and in vivo tissue regeneration. Hence, this study addresses the challenges typical to decellularized ECM bioinks with possibilities to further improve their mechanical strength for long-term stability.

## 4. Conclusions

The main goal of this study was to modify the dLM suitable for bioprinting under truly physiological conditions, i.e., at 37 °C and at a 7–7.4 pH value. Prior to our work, only a few recent studies have started focusing on the importance of bioprinting at physiological temperatures to improve cell functions [52]. It is an important criterion, especially in developing complex advanced constructs that would require all the aforementioned parameters. In this study, a temperature-sensitive dLM bioink was modified to generate a highly crosslinked 3D structure with a cytocompatible gelation process and optimized viscoelasticity suitable for extrusion. The 3D printed construct supported the growth of HepG2 cells and began to display liver-specific functions for a period of 7 days. The current study lays the foundations for the application of highly crosslinked dLM-G-PEG-T for toxicological studies with HepG2 cells. Still, it would benefit further from the investigation of an updated and well-defined protocol for creating a physiologically relevant liver model to mimic the human drug response. Overcoming the limitations of printing conditions would allow 3D models to be acceptable for high-throughput applications with better representation for drug screening and in vitro disease models. This study paves the way for future generations of dLM bioinks to diminish the gaps between 3D biofabrication and its biomedical applications.

## Data Availability

Not applicable.

## Supplementary Materials

The following supporting information can be downloaded at: https://www.mdpi.com/article/10.3390/bios12070521/s1, Table S1: dLM formulations for rheology and TNBS assay with experimental conditions. Figure S2: dLM-sol at room temperature. Figure S3: dLM-G with varying gelatin concentration. Figure S4: Various dLM-G formulations with 10% gelatin. Table S2: 10–12% gelatin to dLM volume ratio to analyze the bioink property. Figure S5: Characterization of bioink with spatula and injection. Figure S6: Frequency sweep of pH-adjusted dLM-sol, dLM-G, dLM-G-PEG, and dLM-G-PEG-T. Table S3: Storage and loss modulus of all the formulations from Table S1 at 37 °C at 1% strain. Figure S7: Scanning electron microscopy image of pH-adjusted dLM-sol (left) and dLM-G-PEG bioink (right). Figure S8: Bioprinting analysis. Figure S9: HepG2 viability study, Figure S10: HpeG2 control study with collagen.
