# Supplementary material for: 3D Bioprinting of Multi-Material Decellularized Liver Matrix Hydrogel at Physiological Temperatures
Source: Biosensors (Basel). 2022 Jul 13;12(7):521. doi: 10.3390/bios12070521 (PMC9313433; doi:10.3390/bios12070521)
Supplement: Supplementary file 1 [file biosensors-12-00521-s001.zip › biosensors-1731746-supplementary.pdf]

biosensors

MDPI

Supplementary Materials

3D Bioprinting of Multi-Material Decellularized Liver Matrix Hydrogel at Physiological Temperatures

Vamakshi Khati [1], Harisha Ramachandraiah [2], Falguni Pati [3], Helene A. Svahn [1], Giulia Gaudenzi [1,4] and Aman Russom [1,5,*]

1 Division of Nanobiotechnology, Science for Life Laboratory, Department of Protein Science, KTH Royal Institute of Technology, 17165 Solna, Sweden; khati@kth.se (V.K.); helene.andersson.svahn@scilifelab.se (H.A.S.); giulia.gaudenzi@ki.se (G.G.); aman@kth.se (A.R.)
2 Biopromic AB, 17165 Solna, Sweden; harishabio@gmail.com
3 Department of Biomedical Engineering, Indian Institute of Technology Hyderabad, Kandi 502285, India; falguni@bme.iith.ac.in
4 Department of Global Public Health, Karolinska Institute, 17165 Solna, Sweden; giulia.gaudenzi@ki.se
5 AIMES—Center for the Advancement of Integrated Medical and Engineering Sciences, Karolinska Institute and KTH Royal Institute of Technology, Sweden
* Correspondence: aman@kth.se

Table S1: dLM formulations for rheology and TNBS assay with experimental conditions

	Formulations	Composition	pH value
a)	dLM-sol	3% solution	3
b)	pH adjusted dLM- sol	3% solution adjusted with NaOH	7–7.4
c)	dLM -G	1:5 dLM to gelatin ratio	7–7.4
d)	dLM- G-PEG	14.4 mg/ml x-PEG-x of 1:5 dLM-G mix	7–7.4
e)	dLM- G-PEG -T	500 U of tyrosinase with 1 ml of dLM-G-PEG	7–7.4

S2: dLM-sol at room temperature

dLM-sol prepared by pepsin digestion of decellularized liver tissue with acetic acid. The solution is prepared in bulk (pH 2-3).

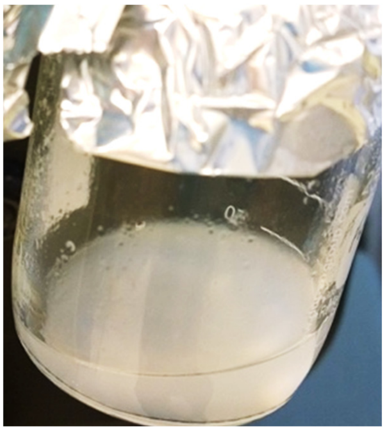

Figure S2. Decellularized liver matrix (dLM-sol) at room temperature.

S3: dLM-G with varying gelatin concentration

dLM-G at 1:5 gelatin to dLM ratio (w/v %) at varying concentrations of gelatin starting with 3% to 8%. A change in the viscosity was observed with the inverted tube. 3% gelatin showed a very liquid-like behavior with dLM which improved when 5% gelatin was used instead. 8% gelatin started to become viscous in the given dLM-G formulation.

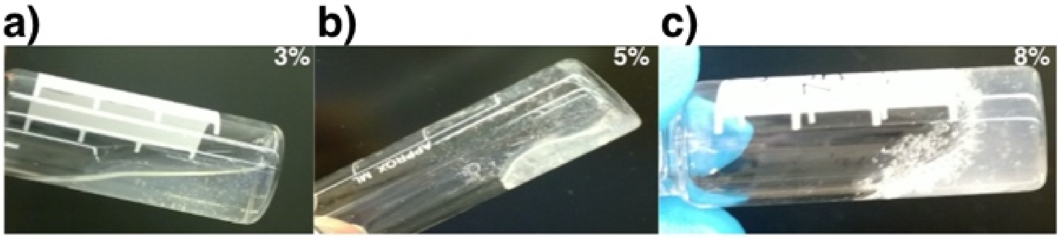

Figure S3. Increasing concentration of gelatin from a) 3%, b) 5%, and c) 8% with improvement in tube inversion behavior.

S4: Various dLM-G formulations with 10-12% gelatin

10% Gelatin to dLM volume ratio of different formulations and their tube inversion behavior. The formulations were designated either 'robust' if they maintained shape or 'soft' if they spread easily and 'brittle' if they broke easily. 1:10 formulation was easily breakable, and its 3D printing attempt was also unsuccessful. 1:4 formulation was robust in nature and the 1:2 formulation was very liquid in nature. Thus a clear behaviour with increasing softness with increasing gelatin volume is observed below.

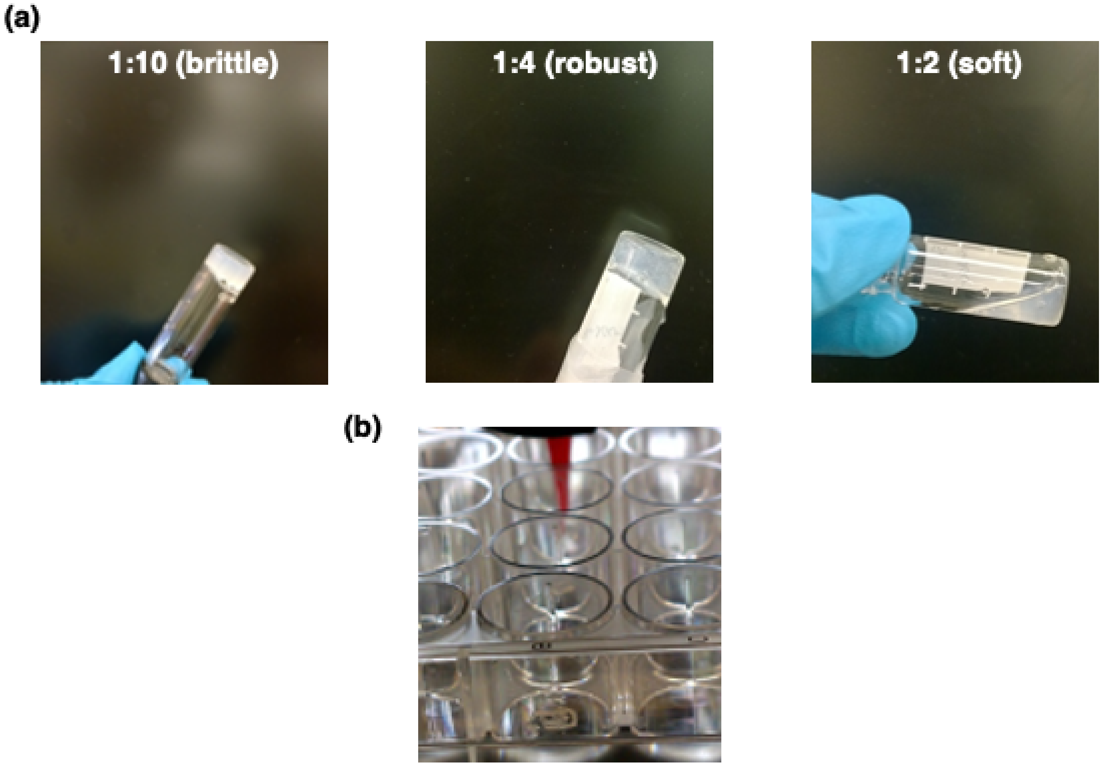

Figure S4. a) 10-12% Gelatin to dLM volume ratio with the lowest concentration of gelatin with brittle nature to highest concentration of gelatin with soft nature. b) 3D printing attempt of 1:10 ratio dLM-G formulation.

Table S2: 10-12% gelatin to dLM volume ratio to analyze the bioink property

Volume ratio	Bioink property
a) 1:10	brittle
b) 1:8	brittle
c) 1:6	robust
d) 1:4	robust
e) 1:2	soft
f) 1:1	soft

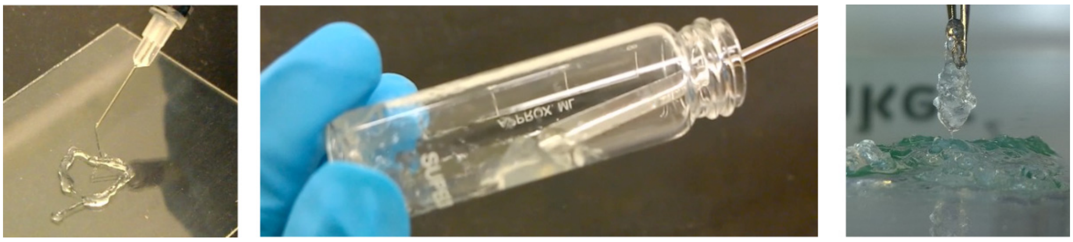

Figure S5. Characterization of bioink with spatula and injection. Visual characterization by inspecting the spreadability of 1:5 formulation of dLM-G-PEG through a spatula and injecting it on a plate.

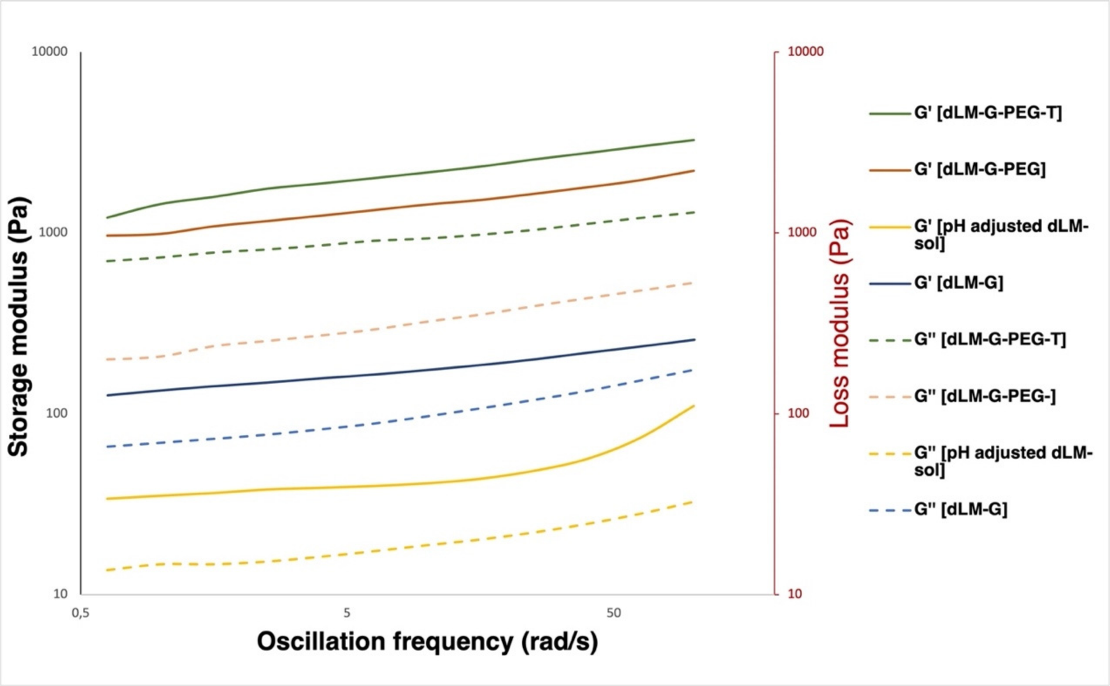

Figure S6. Frequency sweep of pH adjusted dLM-sol, dLM-G, dLM-G-PEG and dLM-G-PEG-T.

Table S3: Storage and loss modulus of all the formulations from Table S1 at 37 °C at 1% strain.

Formulations	Storage modulus G′ (Pa)	Loss modulus G′′ (Pa)
pH adjusted dLM-sol	60	16
dLM-G	242	102
dLM-G-PEG (bioink)	1288	315
dLM-G-PEG-T	1928	685

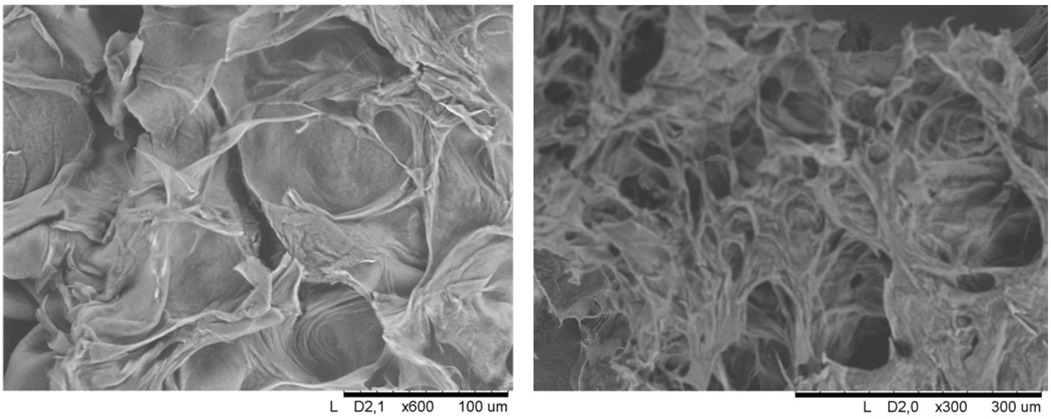

Figure S7. Scanning electron microscopy image of pH adjusted dLM-sol (left) and dLM-G-PEG bioink (right). Reduction in porosity is visible after crosslinking resulting in a tighter and dense bioink structure.

S8: Bioprinting analysis

Bioprinting at 37 °C with filament flow, the filament width, the crosslinked 3D printed consrtuct and long term analysis.

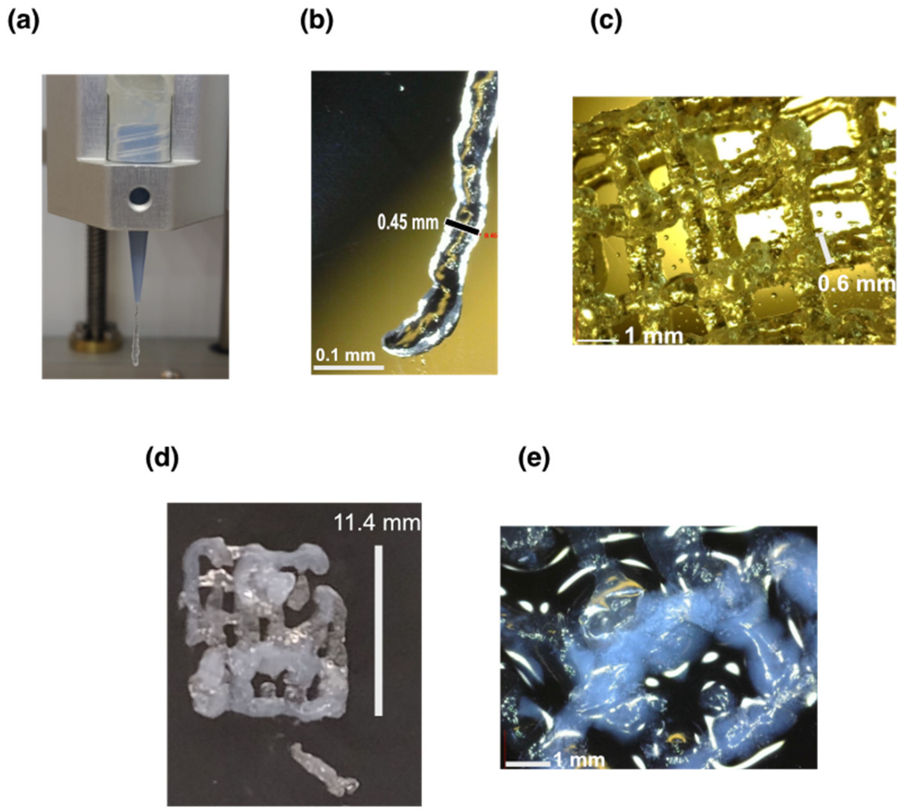

Figure S8. Bioprinting at 37 °C. (a) Filament of dLM-G-PEG extruding with temperature control and (b) the width of the filament. (c) Microscopic image of dLM-G-PEG-T after crosslinking. (e) Visual and (f) microscopic analysis of dLM-G-PEG-T for long term stability at Day 21 (scale bar 100)**.**

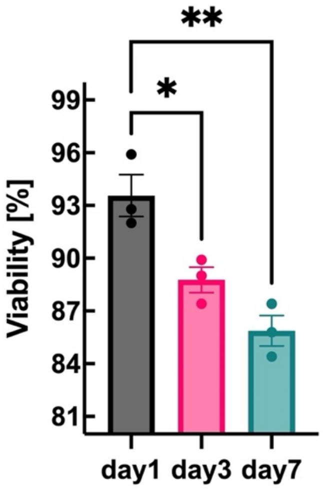

Figure S9. HepG2 viability study. Cell viability within 3D printed dLM-G-PEG-T construct at days 1, 3 and 7 (*$p < 0.05$ and **$p < 0.01$)

S10: HepG2 control study with collagen. HepG2 cells embedded in collagen were used as a control group to compare the cell viability, proliferation and albumin production. Increasing cell viability can be observed in the live/dead assay from day 1 to day 7. Alamar blue assay also showed an increase in cell activity till day 7, however, the albumin assay shows an insignificant increase in albumin production after day 3.

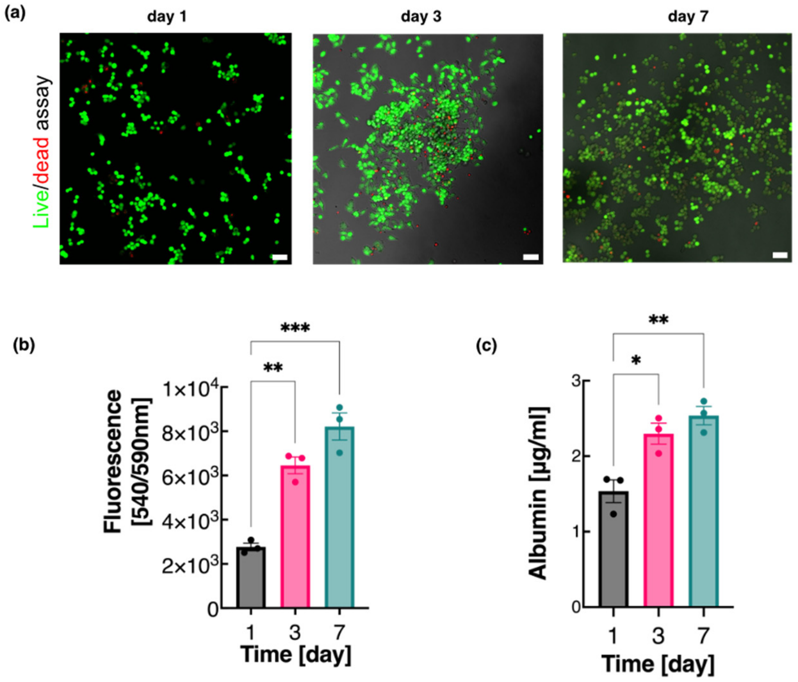

Figure S10. Cell viability within HepG2 embedded collagen control samples. (a) Live/dead assay (scale bar 100 µm), (b) Alamar blue assay, and Albumin production at days 1, 3 and 7. Error bars show the standard error of the mean (**$p < 0.05$, **$p < 0.01$ and ***$p < 0.001$).
